# Furmonertinib combined with bevacizumab in EGFR-TKI-resistant leptomeningeal metastasis: analysis of the CSF ctDNA molecular response and survival outcomes

**DOI:** 10.1038/s41416-026-03407-z

**Published:** 2026-04-06

**Authors:** Xiaoyue Wang, Yuwen Xie, Jin Hu, Na Liu, Liangfeng Yang, Ting Xu, Shu Xu, Chuanyong Yu, Shencun Fang

**Affiliations:** 1 Department of Respiratory Medicine, Nanjing Chest Hospital, The Affiliated Brain Hospital of Nanjing Medical University, Nanjing, China; 2 Brain Metastases Diagnosis and Treatment Centre, The Affiliated Brain Hospital of Nanjing Medical University, Nanjing, China; 3 Department of Respiratory Medicine, Nanjing Gaochun People’s Hospital, Nanjing, China; 4 Department of Neurology, The Affiliated Brain Hospital of Nanjing Medical University, Nanjing, China

**Keywords:** Non-small-cell lung cancer, Medical research

## Abstract

**Background:**

Leptomeningeal metastasis (LM) after the development of third-generation epidermal growth factor receptor tyrosine kinase inhibitor (EGFR-TKI) resistance is indicative of a poor prognosis in EGFR-mutant non-small cell lung cancer (NSCLC), and no standardised treatments are currently available. The aims of this study were to evaluate the outcomes of combination therapy with bevacizumab plus high-dose furmonertinib in this setting and to assess the cerebrospinal fluid (CSF) circulating tumour DNA (ctDNA) molecular response as a treatment response biomarker.

**Methods:**

This real-world study included 104 patients with EGFR-mutant NSCLC who experienced LM progression after treatment with third-generation TKIs. Cohort 1 (*n* = 62) received combination therapy with furmonertinib (160 mg) + bevacizumab, and Cohort 2 (*n* = 42) received furmonertinib (160 mg) monotherapy. The primary endpoints were intracranial progression-free survival (iPFS) and overall survival (OS). In the longitudinal CSF ctDNA analysis, a molecular response was defined as follows: ΔctDNA ≤ 0.8 × baseline.

**Results:**

Combination therapy with bevacizumab plus high-dose furmonertinib significantly improved the LM response compared to that of furmonertinib monotherapy (median iPFS: 6.77 vs 4.04 months, respectively, 95% CI: 0.41–0.98, *p* = 0.038; median OS: 15.31 vs 7.10 months, respectively, 95% CI: 0.29–0.82, *p* = 0.002). The CSF ctDNA analysis revealed that 31/47 patients (66%) achieved a molecular response; those that did experienced significantly prolonged survival outcomes compared to those of patients who did not (iPFS: 8.94 vs 6.67 months, respectively (HR = 0.40, 95% CI: 0.21–0.79); OS: 20.44 vs 8.71 months, respectively (HR = 0.34, 95% CI: 0.14–0.83)). A longitudinal decline in ctDNA across two time points further correlated with survival benefits (iPFS: 9.96 vs 7.33 months (HR = 0.42, *p* = 0.01); OS: 25.63 vs.15.31 months, (HR = 0.28, *p* = 0.03)).

**Conclusion:**

Bevacizumab synergises with high-dose furmonertinib to significantly improve survival outcomes in TKI-resistant LM. A positive CSF ctDNA molecular response (ΔctDNA ≤ 0.8 × baseline) is predictive of clinical benefits, supporting its utility for real-time monitoring. This combination therapy represents a promising strategy for the treatment of a population with unmet needs.

## Introduction

Leptomeningeal metastasis (LM) is a devastating complication of advanced epidermal growth factor receptor (EGFR)-mutant non-small cell lung cancer (NSCLC) that occurs in approximately 10% of patients and is associated with a median overall survival (OS) of only 3.6–11.0 months [1–3]. Although third-generation EGFR tyrosine kinase inhibitors (TKIs) have better penetration of the blood–brain barrier (BBB) and may be initially efficacious in combating LM [4–6], resistance inevitably develops, and there are no standard treatment options currently available for combating LM progression following third-generation TKI failure.

Furmonertinib, a highly brain-penetrant third-generation EGFR-TKI, exhibits potent activity against EGFR-sensitising and resistance-inducing mutations, including the T790M mutation and uncommon variants [7–9]. Both preclinical and clinical research have indicated that furmonertinib penetrates the CNS more efficiently than other EGFR-TKIs, with a brain-to-plasma ratio that is 1.8-fold higher than that of osimertinib [10, 11]. Notably, high-dose furmonertinib (160 mg) has exhibited promising intracranial efficacy in patients experiencing disease progression during treatment with prior third-generation EGFR-TKIs, achieving a median intracranial progression-free survival (iPFS) of 4.3–9.8 months [12, 13]. However, the survival benefits of furmonertinib monotherapy remain suboptimal, underscoring the need for synergistic therapeutic combinations to enhance disease control within the CNS. Preclinical evidence suggests that bevacizumab, a vascular endothelial growth factor (VEGF)-targeting monoclonal antibody, may potentiate EGFR-TKI efficacy by normalising the vasculature of tumours and improving drug delivery to lesions within the CNS [14–17]. Clinically, combination therapy with osimertinib and bevacizumab has been shown to prolong survival in cases involving LM [18]; however, the outcomes of bevacizumab combined with high-dose furmonertinib in patients who have experienced LM after developing third-generation TKI resistance have remained unexplored. Therefore, the aim of this study was to assess this combination therapy in this patient population.

Here, we report the first real-world, retrospective cohort study comparing the efficacy and safety of high-dose furmonertinib combined with bevacizumab versus high-dose furmonertinib monotherapy in patients with EGFR-mutant NSCLC and LM who progressed after prior third-generation EGFR-TKI therapy. Furthermore, the utility of cerebrospinal fluid (CSF) circulating tumour DNA (ctDNA) was explored as a dynamic biomarker, and a molecular response threshold was defined that was capable of predicting survival outcomes in this patient population.

## Materials and methods

### Study design and population

This real-world study analysed data collected from 104 patients with EGFR-mutant NSCLC who experienced LM and developed resistance to third-generation EGFR-TKIs (osimertinib, almonertinib, or furmonertinib) between March 2021 and July 2024. The inclusion criteria were as follows: (1) histologically or cytologically confirmed NSCLC with EGFR-sensitising mutations; (2) LM diagnosis confirmed via CSF cytology (based on the presence of malignant cells) and/or radiographic evidence of leptomeningeal enhancement/ventricular broadening; (3) LM progression following third-generation EGFR-TKI treatment; and (4) treatment with high-dose furmonertinib (160 mg daily) in combination with bevacizumab, requiring two cycles of bevacizumab administration. Exclusion criteria included patients with severe, uncontrolled systemic comorbidities, lack of available follow-up data, or uncontrolled symptomatic brain or leptomeningeal disease. During the same period, a cohort of patients treated with high-dose furmonertinib (160 mg/day) monotherapy (without bevacizumab) was also enrolled according to the same criteria. A flowchart of the study design and selection process is shown in Fig. S1. This study was conducted in accordance with the Declaration of Helsinki and was approved by the Institutional Review Board of Nanjing Chest Hospital (Number: 2023-KL023-02), which waived the requirement for informed consent from the included patients.

### Data collection and response evaluation

Clinical data were obtained from electronic medical record databases and included information on the patients’ demographic characteristics, treatment modalities and clinical outcomes. The clinical response in each case was assessed based on the improvement of neurological symptoms, CSF analyses, radiographic assessments and clinical symptoms evaluated according to the Response Assessment in Neuro-Oncology (RANO) Working Group on LM criteria [19]. LM was radiographically evaluated using gadolinium-enhanced brain magnetic resonance imaging (MRI) in accordance with the EANO–ESMO consensus [20]. Imaging assessments were conducted at baseline and subsequently every 8–12 weeks during treatment, or earlier if clinically indicated (e.g. new or worsening neurological symptoms). Treatment responses were categorised as a positive LM response, stable disease (SD), or progressive disease (PD). The LM ORR was defined as the proportion of patients who achieved a positive LM response. The LM disease control rate (DCR) was defined as the proportion of patients who achieved either an LM response or exhibited SD. iPFS is characterised by the time from the onset of high-dose furmonertinib treatment to either the advancement of intracranial disease or death, depending on which happens first. OS was described as the duration from the start of high-dose furmonertinib treatment until death from any reason. Safety was assessed based on the occurrence of adverse events, assessed according to the Common Terminology Criteria Adverse Events version 5.0 (CTCAE 5.0).

### Genomic profiling

Following the LM diagnosis, CSF samples (volume, ~10 mL) were collected. Longitudinal CSF samples were collected during routine clinical follow-up. The follow-up sample was typically obtained, coinciding with the clinical and radiographic evaluation. Cell-free DNA was extracted using a QIAamp Circulating Nucleic Acid Kit (QIAGEN, Germany) and quantified using a double-stranded DNA (dsDNA) HS Assay Kit (Thermo Fisher Scientific, USA). Target enrichment was performed using a 1,012-gene probe panel (Yucebio, China) for hybridisation capture. Libraries were subjected to paired-end sequencing (100 base pairs) on a DNBSEQ-T7 platform. Bioinformatics processing involved three main steps. First, for read filtering and alignment, fastp (v0.20.0) software was used to remove low-quality reads, and BWA-MEM was used to align clean reads to hg19. Second, for UMI processing and variant calling, Gencore (v0.14.0) was used to merge the reads with shared UMIs, and VarDict was used to identify single-nucleotide variants and insertions/deletions (indels). Third, for annotation and filtering, the variants were annotated using the Ensembl Variant Effect Predictor (VEP v101), and subsequent filtering was performed to exclude both variants with a variant allele frequency <0.02 as well as known germline mutations. The change in ctDNA (ΔctDNA) was calculated as previously described [21], by dividing the on-treatment ctDNA concentration measured at the second monitoring timepoint by the baseline ctDNA concentration within a cohort. For patients with undetectable ctDNA at baseline, the cohort-specific limit of detection served as a substitute concentration value.

### Statistical analysis

The demographic and clinical characteristics of the patients, as well as the safety data and response rates, were summarised using descriptive statistics. For the comparison of continuous variables between independent groups, the Wilcoxon rank-sum test was applied. ORR and DCR with 95% confidence intervals (CIs) were computed based on the exact binomial distribution (Clopper–Pearson method). Survival outcomes (iPFS and OS) were evaluated using Kaplan–Meier curves and log-rank tests. Hazard ratios (HRs) and 95% CIs were calculated using Cox proportional hazards models after adjusting for baseline covariates. Variables with *p* values < 0.05 in the univariate analyses were incorporated into the multivariate models. Statistical significance was defined as a two-sided *p* value < 0.05. All statistical analyses and graphing were performed using SPSS Statistics version 26 and GraphPad Prism 8 software.

### Ethics approval and consent to participate

The study was performed according to the ethical guidelines of the Declaration of Helsinki and was approved by the ethics committee of Nanjing Chest Hospital (Number: 2023-KL023-02).

## Results

### Patient characteristics

A total of 104 patients with EGFR-mutant NSCLC, who developed resistance to third-generation EGFR-TKIs, participated in this real-world study. Patients were categorised into the following two groups: Cohort 1 received high-dose furmonertinib (160 mg daily) combined with bevacizumab, and Cohort 2 received high-dose furmonertinib monotherapy. Baseline demographic and clinical characteristics were well-balanced between the two cohorts, with no statistically significant differences (*p* > 0.05 for all variables; Table 1). The median age was 57 years (range: 31–77 years) in both cohorts. Most patients were female (62.9% in Cohort 1 vs. 61.9% in Cohort 2), and all of them (100%) had histologically confirmed adenocarcinoma. In Cohort 1, 33.9% of the patients had an Eastern Cooperative Oncology Group (ECOG) performance status (PS) of 2, whereas 66.1% had an ECOG PS of 3–4, and the distributions were comparable between the two cohorts (*p* = 0.78). The EGFR exon 21 L858R mutation was the predominant mutation (58.1% in Cohort 1 vs. 45.3% in Cohort 2; *p* = 0.43). In the overall population prior to LM diagnosis, the following third-generation EGFR-TKIs were administered: 63.5% received osimertinib, 43.3% were treated with almonertinib and 24% had been prescribed furmonertinib. Despite the development of LM, extracranial disease remained stable in most patients (90.3% in Cohort 1 vs. 85.7% in Cohort 2). During LM treatment, most patients received intrathecal chemotherapy (82.3% in Cohort 1 vs. 78.6% in Cohort 2), whereas whole-brain radiotherapy (WBRT) was administered to 30.6% and 28.6% of the patients, respectively.

**Table 1:** Patient characteristics.

Characteristics	Furmonertinib + Bev(%) (*N* = 62)	Furmonertinib(%) (*N* = 42)	*P*
Age in years, median (range)	57 (31–77)	57 (34–77)	
<60, *n* (%)	39 (62.9)	29 (69.0)	0.52
≥60, *n* (%)	23 (37.1)	13 (31.0)	
Sex, *n* (%)			
Male	23 (37.1)	16 (38.1)	0.92
Female	39 (62.9)	26 (61.9)	
Histological type, *n* (%)			
Adenocarcinoma	62 (100)	42 (100)	NA
ECOG PS *n* (%)			
2	21 (33.9)	17 (40.5)	0.78
3	22 (35.5)	13 (30.9)	
4	19 (30.6)	12 (28.6)	
Smoking status, *n* (%)			
Smoker	13 (21.0)	14 (33.3)	0.16
Nonsmoker	49 (79.0)	28 (66.7)	
EGFR mutation, *n* (%)			
EGFR L858R	36 (58.1)	19 (45.3)	0.43
EGFR-19del	15 (24.2)	14 (33.3)	
Other EGFR mutations	11 (17.7)	9 (21.4)	
Concurrent BM, *n* (%)			
Yes	28 (45.2)	18 (42.9)	0.82
No	34 (54.8)	24 (57.1)	
Lines of treatment, *n* (%)			
≤3	32 (51.6)	26 (61.9)	0.30
>3	30 (48.4)	16 (38.1)	
Classification of extracranial lesion after LM, *n* (%)			
SD	56 (90.3)	36 (85.7)	0.47
PD	6 (9.7)	6 (14.3)	
Previous third-generation EGFR-TKI therapy, *n* (%)			
Osimertinib	37 (59.8)	29 (69.0)	0.68
Almonertinib	29 (46.8)	16 (38.1)	
Furmonertinib	15 (24.2)	10 (23.8)	
Intrathecal chemotherapy, *n* (%)			
Yes	51 (82.3)	33 (78.6)	0.64
No	11 (17.7)	9 (21.4)	
WBRT, *n* (%)			
Yes	19 (30.6)	12 (28.6)	0.82
No	43 (69.4)	30 (71.4)	

*Bev* bevacizumab, *ECOG PS* Eastern Cooperative Oncology Group Performance Status, *EGFR* epidermal growth factor receptor, *LM* leptomeningeal metastasis, *SD* stable disease, *PD* progressive disease, *TKI* tyrosine kinase inhibitor, *BM* brain metastasis, *WBRT* whole-brain radiotherapy.

### Clinical outcomes of high-dose furmonertinib plus bevacizumab

**Fig. 1:**
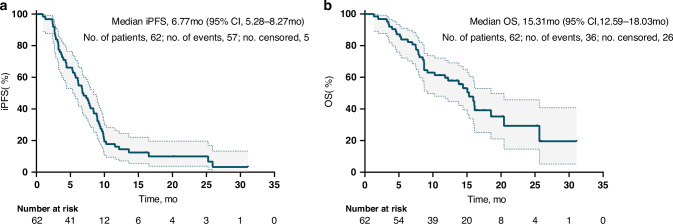
Kaplan–Meier curves of survival analysis in patients with NSCLC who experienced LM. iPFS (**a**) and OS (**b**). iPFS intracranial progression-free survival, OS overall survival, NSCLC non-small cell lung cancer, LM leptomeningeal metastasis.

**Fig. 2:**
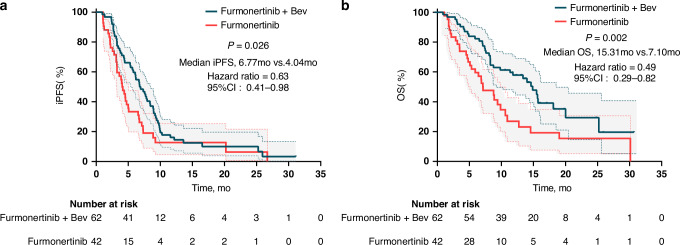
Kaplan–Meier comparative survival analysis between patients treated with high-dose furmonertinib plus bevacizumab combination therapy and high-dose furmonertinib monotherapy. **a** Intracranial progression-free survival (iPFS). **b** Overall survival (OS).

In terms of treatment responses in Cohort 1, 34 patients (54.8%) demonstrated an LM response, and 21 (33.9%) had SD, yielding an LM ORR of 54.8% (95% CI: 41.7–67.5) and a DCR of 88.7% (95% CI: 78.1–95.3). In Cohort 2, these proportions were 21.4% (9/42) and 57.2% (24/42) respectively, resulting in an ORR of 21.4% (95% CI: 10.3–36.8) and a DCR of 78.6% (95% CI: 63.2–89.7) (Table 2). At the data cutoff time (December 2024), the median duration of the follow-up period was 8.3 months (range: 0.8–28.2 months), and 91.9% (57/62) of patients experienced iPFS events. In Cohort 1, the median iPFS reached 6.77 months (95% CI: 5.28–8.27, Fig. 1a), and the median OS was 15.31 months (95% CI: 12.59–18.03, Fig. 1b). For Cohort 2, the median iPFS was 4.04 months (95% CI: 3.07–5.01), and the median OS was 7.10 months (95% CI: 4.29–9.91). In Cohort 1, treatment with the combination regimen significantly improved the survival outcomes compared to those of high-dose furmonertinib monotherapy (median iPFS: 6.77 vs 4.04 months, respectively (HR: 0.63, 95% CI: 0.41–0.98, *p* = 0.026, Fig. 2a); median OS: 15.31 vs 7.10 months, respectively (HR: 0.49, 95% CI: 0.29–0.82, *p* = 0.002, Fig. 2b)).

**Table 2:** Comparison of CNS treatment responses and prognosis in patients with LM.

Analysis set/response	Furmonertinib+Bev (*n* = 62)	Furmonertinib (*n* = 42)
Response, *n* (%)	34 (54.8)	9 (21.4)
SD, *n* (%)	21 (33.9)	24 (57.2)
PD, *n* (%)	7 (11.3)	9 (21.4)
ORR, % (95% CI)	54.8 (41.7–67.5)	21.4 (10.3–36.8)
DCR, % (95% CI)	88.7 (78.1–95.3)	78.6 (63.2–89.7)
iPFS, median (95% CI)	6.77 (5.28–8.27)	4.04 (3.007–5.01)
6-month PFS rate, % (95% CI)	59.7 (46.4–70.7)	33.3 (19.8–47.4)
12-month PFS rate, % (95% CI)	16.1 (8.3–26.2)	12.7 (4.5–25.3)
OS, median (95% CI)	15.31 (12.59–18.03)	7.10 (4.29–9.91)
1-year OS rate, % (95% CI)	59.6 (46.4–70.6)	26.9 (13.3–42.6)
2-year OS rate, % (95% CI)	29.4 (14.7–45.8)	15.4 (5.2–30.6)

*CNS* central nervous system, *iPFS* intracranial progression-free survival, *OS* overall survival, *LM* leptomeningeal metastasis, *Bev* bevacizumab, *SD* stable disease, *PD* progressive disease, *ORR* objective response rate, *CI* confidence interval, *DCR* disease control rate.

**Fig. 3:**
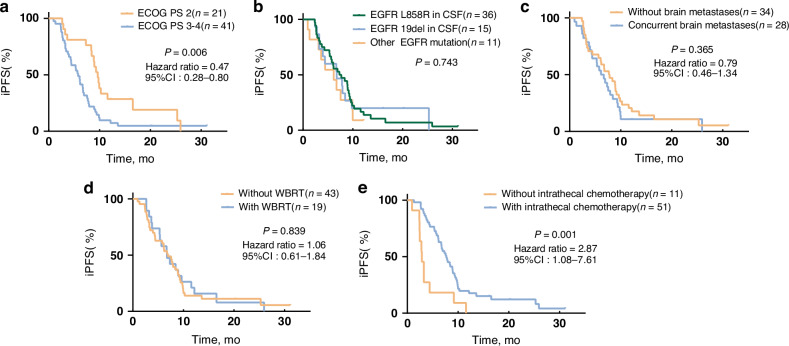
Kaplan–Meier analysis showing the iPFS of patients in different categories. **a** According to ECOG PS (2 or 3–4). **b** According to the EGFR mutation type in CSF (EGFR-21 L858R, EGFR-19del, and other EGFR mutations (uncommon/composite)). **c** With or without brain metastases. **d** With or without WBRT. **e** With or without intrathecal chemotherapy. iPFS intracranial progression-free survival, ECOG PS Eastern Cooperative Oncology Group Performance Status, EGFR epidermal growth factor receptor, WBRT whole-brain radiotherapy.

**Fig. 4:**
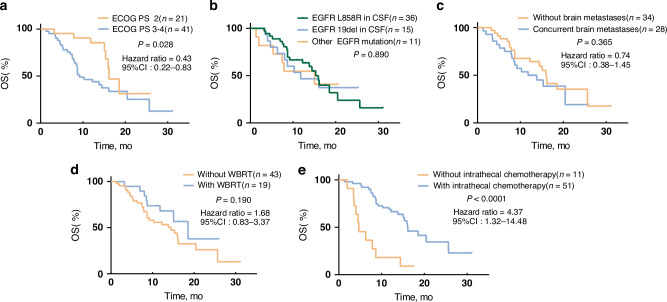
Kaplan–Meier analysis showing the OS of patients in different categories. **a** According to ECOG PS (2 or 3–4. **b** According to the EGFR mutation type in CSF (EGFR-21 L858R, EGFR-19del, and other EGFR mutations (uncommon/composite)). **c** With or without concurrent brain metastases. **d** With or without WBRT. **e** With or without intrathecal chemotherapy. OS overall survival, ECOG PS Eastern Cooperative Oncology Group Performance Status, EGFR epidermal growth factor receptor, WBRT whole-brain radiotherapy.

The subgroup analyses for Cohort 1 revealed comparable median iPFS and OS across groups, irrespective of the WBRT status, presence of brain metastases, or EGFR mutation type (L858R, 19del, or others) (Figs. 3 and 4). Patients with a baseline ECOG PS of 2 had longer iPFS and OS compared to that of patients with an ECOG PS of 3–4 (median iPFS: 9.63 vs. 5.78 months, respectively (HR: 0.47, 95% CI: 0.28–0.80, *p* = 0.006); median OS: 16.13 vs. 9.20 months, respectively (HR: 0.43, 95% CI: 0.22–0.83, *p* = 0.028)). In patients with LM, not receiving intrathecal therapy resulted in significantly shorter survival outcomes compared to those in patients who did receive it (median iPFS: 2.86 vs. 7.69, respectively (HR: 2.87, 95% CI: 1.08–7.61, *p* = 0.001); median OS: 4.67 vs. 16.07, respectively (HR: 4.37, 95% CI: 1.32–14.48, *p* < 0.0001)).

### Univariate and multivariate analyses

The results of the univariate and multivariate analyses of factors related to iPFS in Cohort 1 are shown in Table 3. Both the baseline ECOG PS and intrathecal therapy had a significant impact on iPFS (all *p* < 0.05). The subsequent multivariate Cox regression analysis, which was conducted based on the factors that demonstrated statistical significance in the univariate analysis, confirmed that both the baseline ECOG PS (*p* = 0.007) and the combination of intrathecal therapy (*p* = 0.002) significantly correlated with improved iPFS. Similarly, in the univariate and multivariate analyses of OS (Table 4), baseline ECOG PS (*p* = 0.026) and the combination of intrathecal therapy (*p* < 0.0001) were independent risk factors.

**Table 3:** Univariate and multivariate analysis of iPFS in patients with LM (*n* = 62).

Variables	Univariate		Multivariate	
	HR (95% CI)	*P*	HR (95% CI)	*P*
Age (<60 vs. ≥60 years)	0.662 (0.384–1.141)	0.138		
Sex (male vs. female)	0.815 (0.475–1.399)	0.459		
*TP53* mutation in CSF	0.865 (0.494–1.517)	0.613		
(yes vs. no)				
Smoking status (yes vs. no)	0.995 (0.524–1.892)	0.988		
ECOG PS (2 vs.3–4)	0.455 (0.257–0.805)	0.007	0.453 (0.255–0.805)	0.007
Concurrent brain metastases	1.274 (0.754–2.154)	0.366		
Concurrent bone metastases	1.728 (0.952–3.139)	0.072		
Treatment lines (≤3 vs. >3)	1.098 (0.649–1.857)	0.728		
**Treatments of LM**				
WBRT (yes vs. no)	0.944 (0.539–1.653)	0.839		
Intrathecal chemotherapy	0.335 (0.171–0.659)	0.002	0.335 (0.170–0.660)	0.002
(yes vs. no)				

*iPFS* intracranial progression-free survival, *LM* leptomeningeal metastasis, *HR* hazard ratio, *CI* confidence interval, *TP53* tumour protein p53 gene, *CSF* cerebrospinal fluid, *ECOG PS* Eastern Cooperative Oncology Group Performance Status, *WBRT* whole-brain radiotherapy.

**Table 4:** Univariate and multivariate analysis of OS in patients with LM (*n* = 62).

Variables	Univariate		Multivariate	
	HR (95% CI)	*P*	HR (95% CI)	*P*
Age (<60 years vs. ≥60 years)	0.821 (0.412–1.635)	0.574		
Sex (male vs. female)	0.711 (0.493–1.026)	0.068		
*TP53* mutation in CSF	0.842 (0.426–1.666)	0.622		
(yes vs. no)				
Smoking status (yes vs. no)	0.618 (0.256–1.493)	0.285		
ECOG PS (2 vs.3–4)	0.425 (0.193–0.935)	0.033	0.405 (0.183–0.896)	0.026
Concurrent brain metastases	1.352 (0.701–2.610)	0.368		
Concurrent bone metastases	1.860 (0.771–4.490)	0.168		
Treatment lines (≤3 vs. >3)	0.948 (0.491–1.832)	0.874		
**Treatments of LM**				
WBRT (yes vs. no)	0.594 (0.270–1.306)	0.195		
Intrathecal chemotherapy	0.211 (0.099–0.452)	≤0.0001	0.200 (0.092–0.433)	≤0.0001
(yes vs. no)				

*OS* overall survival, *LM* leptomeningeal metastasis, *HR* hazard ratio, *CI* confidence interval, *TP53* tumour protein p53 gene, *CSF* cerebrospinal fluid, *ECOG PS* Eastern Cooperative Oncology Group Performance Status, *WBRT* whole-brain radiotherapy.

### Identification of ctDNA mutations and analysis of changes

**Fig. 5:**
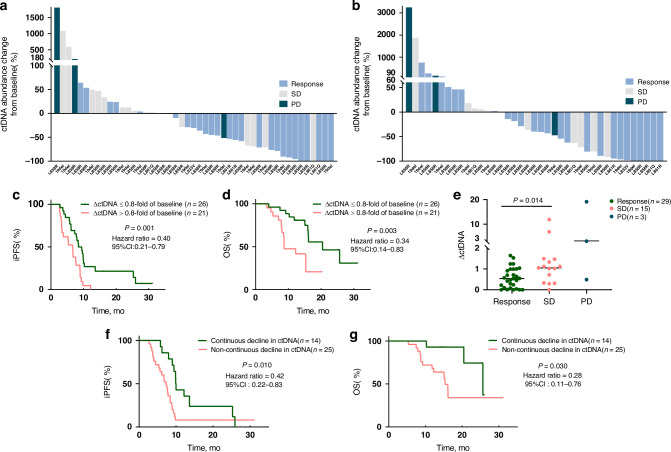
CSF ctDNA analysis. **a**, **b** Waterfall plots showing the percentage change from baseline in the EGFR-mutant ctDNA abundance in the second or third CSF analysis (*n* = 47 and *n* = 39, respectively). **c**, **d** Kaplan–Meier curves of iPFS and OS for the percentage change in ctDNA abundance from baseline in the second CSF analysis (*n* = 47). **e** ΔctDNA values across different treatment response groups. **f**, **g** Kaplan–Meier curves of iPFS and OS for continuous or non-continuous decline in the three CSF analyses (*n* = 39). CSF cerebrospinal fluid, EGFR epidermal growth factor receptor, ctDNA circulating tumour DNA, iPFS intracranial progression-free survival, OS overall survival.

Baseline CSF detection of EGFR-mutant ctDNA occurred in 54 of the 62 eligible Cohort 1 patients with LM upon enrolment. Of these 54 patients, 47 (87%) underwent serial CSF monitoring twice during treatment, and 39 (72.2%) underwent monitoring three times. Figure 5a, b illustrates the longitudinal ctDNA dynamics, quantified as the percentage change from baseline, and the corresponding intracranial response profiles. A reduced ctDNA abundance correlated with a clinical response or SD, whereas an increased abundance corresponded to SD or PD. A 20% reduction in ctDNA concentration from baseline (ΔctDNA ≤ 0.8 × baseline) was established as the threshold for a ‘ctDNA molecular response’. Patients achieving this response demonstrated significantly improved clinical outcomes compared to those in patients who did not (median iPFS: 8.94 vs. 6.67 months, respectively (HR: 0.4, 95% CI: 0.21–0.79); median OS: 20.44 vs. 8.71 months, respectively (HR: 0.34, 95% CI: 0.14–0.83)), as shown in Fig. 5c, d. Moreover, as shown in Fig. 5e, patients who achieved a response demonstrated significantly reduced ΔctDNA levels compared to those in patients with SD (*p* = 0.014). A longitudinal decline in CSF EGFR-mutant ctDNA across two time points correlated with superior iPFS (9.96 vs. 7.33 months, HR: 0.42, 95% CI: 0.22–0.83, *p* = 0.01, Fig. 5f) and OS (25.63 vs.15.31 months, HR: 0.28, 95% CI: 0.11–0.76, *p* = 0.03, Fig. 5g).

### Long-term survival achieved with CSF ctDNA-guided therapy for LM

**Fig. 6:**
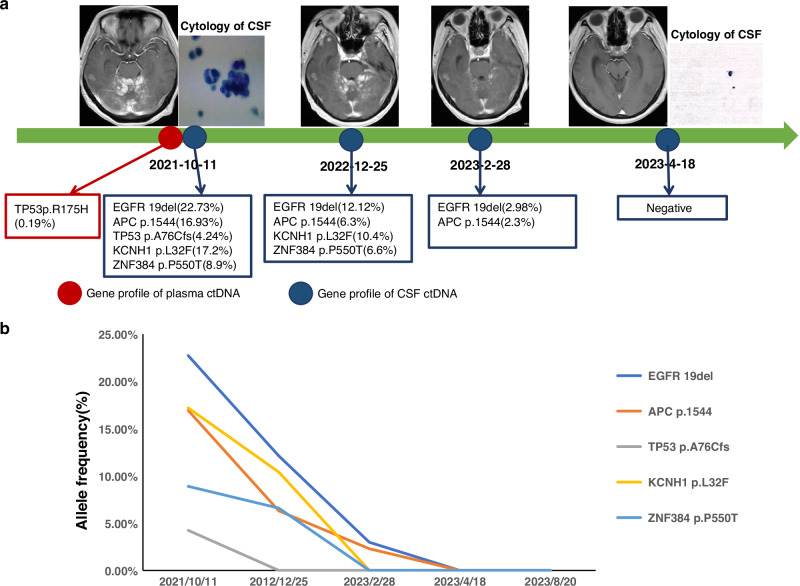
MRI scans, CSF cytology and ctDNA analysis of patients with LM with failed treatment responses to third-generation EGFR-TKIs at different clinical time points. **a** Dynamic changes in MRI scans, CSF cytology and ctDNA. **b** Dynamic changes in the allele frequency of various mutations detected in serial CSF samples. MRI magnetic resonance imaging, CSF cerebrospinal fluid, ctDNA circulating tumour DNA, LM leptomeningeal metastasis, EGFR epidermal growth factor receptor, TKI tyrosine kinase inhibitor.

A 58-year-old woman with advanced EGFR exon 19del-mutant lung adenocarcinoma (diagnosed in 2018) developed LM in October 2021 after experiencing progression while receiving gefitinib-, platinum- and osimertinib-based therapies. Symptomatic presentations (headache, vomiting, visual impairment and paralysis) coincided with MRI-confirmed diffuse leptomeningeal enhancement and CSF cytology-positive LM. CSF/plasma sequencing confirmed the presence of persistent EGFR exon 19del. The patient received intrathecal pemetrexed plus third-line furmonertinib (160 mg/day) and bevacizumab (7.5 mg/kg q3w), which led to the complete resolution of the neurological symptoms and CSF tumour cells (Fig. 6a). A sustained PFS of >26 months was achieved with correlative CSF ctDNA clearance (Fig. 6b).

### Safety

Treatment-related adverse events (TRAEs) occurred in 49/62 patients (79%; Table S1) and were predominantly classified as grade 1–2. The most frequent TRAEs were rashes (14/62, 22.6%), diarrhoea (7/62, 11.3%) and vomiting (5/62, 8.1%). TRAEs of grade ≥3 were observed in eight patients (12.9%) and primarily comprised vomiting, rashes, diarrhoea and nausea. Furmonertinib dose reduction was required in three cases, with one case of treatment discontinuation owing to grade 3 vomiting.

## Discussion

The findings of this real-world study provide compelling evidence that bevacizumab administration can significantly enhance the intracranial efficacy of high-dose furmonertinib in patients with EGFR-mutant NSCLC who have experienced LM progression following third-generation EGFR-TKI failure. The median iPFS and OS for combination therapy were 6.77 months and 15.31 months, respectively, both statistically superior to the survival outcomes of high-dose fruquintinib monotherapy (iPFS: 4.04 months; OS: 7.10 months; *p* < 0.05). These findings highlight the synergistic potential of bevacizumab in overcoming BBB limitations and improving survival in a population of patients with historically dismal outcomes.

The mechanisms underlying LM progression following third-generation EGFR-TKI therapy remain poorly understood. A recent study analysing NGS data from intracranial and extracranial tumours found that CNS metastases were associated with a higher frequency of CARD11 amplification and a lower frequency of MDM2 amplification, extending beyond single-gene alterations [22]. However, pharmacological resistance attributed to inadequate CNS drug penetration is supported by studies that have demonstrated the presence of persistent EGFR mutations in CSF following TKI failure [23–25]. Moreover, the present study found that the extracranial lesions remained stable in most of the patients with LM (90.2% in Cohort 1 and 85.7% in Cohort 2), providing further support for this potential mechanism. Finally, our previous findings also corroborate this possibility, as no established mechanisms of resistance to third-generation TKIs, including those involving either EGFR-dependent or EGFR-independent pathways, have been identified in the CSF genomic profiling of LM cases following third-generation TKI failure [23]. Therefore, enhancing the delivery of drugs via the CSF is a critical strategy. Furmonertinib, a brain-penetrant third-generation EGFR-TKI, overcomes these limitations through structural modifications that enhance its lipophilicity and ability to cross the BBB [10, 26]. Preclinical data have confirmed its superior brain uptake and activity against osimertinib-resistant clones, likely owing to its active metabolite, AST5902, which retains the potency without cross-resistance [27]. These properties align with clinical observations of the efficacy of high-dose furmonertinib in combating TKI-resistant LM, reinforcing the importance of its role in the management of CNS metastases.

This study demonstrated that the combination of high-dose furmonertinib and bevacizumab achieved superior efficacy in heavily pretreated patients with EGFR-mutant NSCLC who developed LM. This was consistent with the findings of a previous study that demonstrated that combining EGFR-TKIs with bevacizumab resulted in a synergistic effect that was mediated by the modulation of E-cadherin levels and an increase in EGFR-TKI concentrations in the brain [18]. This synergistic effect may be attributed to normalisation of the tumour vasculature by bevacizumab, enhancement of BBB permeability to facilitate furmonertinib penetration, and a reduction in VEGF-mediated cerebral oedema [14, 28]. Furthermore, structural modifications of furmonertinib conferred enhanced lipophilicity and brain exposure (1.79× higher than that of osimertinib) (Fig. S2), whereas treatment with its active metabolite AST5902 overcame osimertinib resistance in vitro (Fig. S3). Finally, in this study, 82.3% of patients received intrathecal chemotherapy via the Ommaya reservoir, which ensured direct CSF drug delivery and rapid clearance of malignant cells, and was complementary to systemic therapy [29–32]. Our previous study demonstrated that intrathecal chemotherapy via the Ommaya reservoir not only quickly relieved central symptoms but also greatly prolonged the survival time of patients with NSCLC who developed LM [23, 33, 34]. Collectively, these findings highlight bevacizumab-enhanced furmonertinib therapy as a crucial strategy for managing LM after third-generation TKI failure. Prospective trials are warranted to validate this combination therapy and refine the patient selection criteria.

Several novel agents demonstrate promising intracranial efficacy in advanced NSCLC after EGFR-TKI treatment failure. In the Phase 3 trial, sacituzumab tirumotecan (sac-TMT) significantly improved median PFS (8.3 vs. 4.3 months) compared to chemotherapy in advanced NSCLC patients with disease progression after EGFR-TKI therapy, including those with baseline brain metastases [35]. Additionally, amivantamab–lazertinib exhibited intracranial activity, with iPFS extended to approximately 12.8 months versus 8.3 months with chemotherapy alone in patients with brain metastases [36]. However, while these novel drugs show some activity against brain metastases, their efficacy in LM following resistance to third-generation TKIs remains unknown. In this context, the current study preliminarily demonstrates that high-dose furmonertinib combined with bevacizumab has considerable efficacy in this specific patient population.

The longitudinal analysis of CSF-derived ctDNA in patients with EGFR-mutant NSCLC who developed LM demonstrated the significant prognostic utility of dynamic ctDNA monitoring. The strong correlation between EGFR-mutant ctDNA reduction and improved survival outcomes establishes CSF ctDNA as a real-time biomarker of treatment responses in this challenging patient population. Critically, we proposed a novel ‘ctDNA molecular response’ threshold (ΔctDNA ≤0.8-fold baseline), which was able to effectively identify patients with markedly superior iPFS (8.94 vs. 6.67 months; HR: 0.4) and OS (20.44 vs. 8.71 months; HR: 0.34). This threshold surpassed radiographic assessments in predicting survival outcomes and aligned with emerging evidence that liquid biopsy may be useful in supplementing the traditional response criteria for CNS metastases [37, 38]. The observed discordance in some patients with SD, in whom rising ctDNA levels preceded radiographic progression, suggests that CSF ctDNA dynamics may serve as an early indicator of treatment resistance. This is consistent with the findings of studies conducted by Bai et al., which showed that CSF ctDNA clearance correlated with EGFR-TKI efficacy in patients with LM [25]. The present study built on this concept by quantifying the magnitude of ctDNA reduction required for meaningful survival benefits, thereby providing a clinically actionable benchmark.

This study had some limitations that warrant consideration when interpreting the findings. First, the retrospective design meant that there was an inherent risk of selection bias, particularly in terms of the LM diagnosis and response assessment, despite the adherence to RANO criteria. Second, the heterogeneity in prior therapies and non-standardised intrathecal chemotherapy regimens may have served as confounders that affected the survival outcomes that were attributed to the combination of bevacizumab and furmonertinib. Third, while the longitudinal assessment of CSF ctDNA dynamics demonstrated strong prognostic utility, incomplete longitudinal sampling (47/54 patients with ≥2 time points; 39/54 with ≥3) limited the uniform application of the proposed molecular response threshold. These constraints do not diminish the primary conclusion that bevacizumab synergises with high-dose furmonertinib to improve survival in TKI-resistant LM, although they do highlight the need for prospective validation in controlled settings.

## Conclusion

Bevacizumab synergises with high-dose furmonertinib to significantly improve the survival of patients with EGFR-mutant NSCLC who develop TKI-resistant LM. The CSF ctDNA molecular response (defined as a ΔctDNA ≤ 0.8 × baseline) can serve as a dynamic biomarker capable of predicting superior survival outcomes. This combination therapy addresses an urgent unmet need in LM management, while dynamic ctDNA monitoring facilitates more precise intervention.

## Supplementary information

Supplementary Methods and Table

Flowchart of the screening procedure.

Comparative pharmacokinetics of furmonertinib and Osimertinib.

Furmonertinib and its active metabolites showed no cross-resistance with Osimertinib.

## Data Availability

All data relevant to the study are included in this article. Further inquiries can be directed to the corresponding author.
