# Supplementary material for: Furmonertinib combined with bevacizumab in EGFR-TKI-resistant leptomeningeal metastasis: analysis of the CSF ctDNA molecular response and survival outcomes
Source: Br J Cancer. 2026 Apr 6;134(11):1614–23. doi: 10.1038/s41416-026-03407-z (PMC13184092; doi:10.1038/s41416-026-03407-z)
Supplement: Supplementary file 1 — Supplementary Methods and Table [file 41416_2026_3407_MOESM1_ESM.docx]

## Furmonertinib Combined with Bevacizumab in EGFR-TKI-Resistant Leptomeningeal

## Metastasis: Analysis of the CSF ctDNA Molecular Response and Survival Outcomes

## Supplementary Methods

**Establishment of osimertinib-resistant cell line**

To establish osimertinib-resistant sublines, parental PC9 cells were subjected to the osimertinib adaptation protocol. Briefly, cells were initially exposed to 10 nM osimertinib for two weeks, followed by prolonged culture in 100 nM of osimertinib for four weeks, and then further cultured in 500 nM of osimertinib for another two weeks, finally generating the PC9/OR cell line. For resistance validation, PC9 and PC9/OR cells were seeded at 10,000 cells/well in 96-well plates, and challenged with osimertinib concentrations of 10, 100, 1000, and 5000 nM for 48 h, followed by cytotoxicity assessment via a Cell Counting Kit-8.

**In vitro cell growth inhibition assay**

PC9 and PC9/OR cells were exposed to a series of concentrations of osimertinib (10, 100, 1000, and 5000 nM), furmonertinib (10, 100, 1000, and 5000 nM), or AST5902 (10, 100, 1000 and 5000 nM) for 72 h at 37 ℃ with 5% CO_2_. After treatment, cell viabilities were measured using a CCK-8 Assay Kit and quantified by comparing the resultant viabilities with those in the absence of drug exposure. IC50 values were calculated via GraphPad Prism 9 (GraphPad Software Inc., San Diego, CA, USA).

**Pharmacokinetics study**

Pharmacokinetics profiles of furmonertinib and osimertinib *in vivo* were analyzed in mice models. All animal care and experimental procedures complied with the National Research Council's Guide for the care and use of laboratory animals and animal welfare and were approved by the SPF Animal Laboratory of China Pharmaceutical University (animal authorization reference number: SYXK2021-0011). Healthy female Balb/c nude mice (aged 4-5 weeks, weighing 16-18 g) were obtained from the Beijing Vital River Laboratory Animal Technology Co., Ltd. (Beijing, China). Mice were randomly assigned into two groups and treated with furmonertinib (20 mg/kg, i.g.) and osimertinib (20 mg/kg, i.g.) respectively. Blood samples (50 μL) were collected from the tail vein at different time points after drug administration (blood samples were collected no more than three times per mouse). The concentrations of furmonertinib, osimertinib, and their active metabolites in plasma and brain were determined by liquid chromatography-tandem mass spectrometry, as detailed in the Supplementary Material.

**Liquid chromatography-tandem mass spectrometry assay**

The concentrations of Furmonertinib, Osimertinib and their active metabolites in plasma and brain were prepared and analyzed on a Shimadzu LC-20 HPLC system (Kyoto, Japan) coupled to an MS/MS system (SCIEX, Birmingham, MA, USA). Briefly, samples were protein-precipitated with 4 times the volume of ice-cold methanol containing 200 ng/mL Almonertinib (Internal standard, IS). After centrifugation (30,000 g, 10 min, 4 ℃), the supernatant was collected and injected into the LC-MS/MS system for analysis. Chromatographic separation was performed on a Luna C18 column (150 × 2.0 mm, 5 μm, Phenomenex, USA) at 40 ℃. The mobile phase consisted of solvent A (0.1% acetic acid and 5 mM ammonium acetate) and solvent B (acetonitrile) with the following gradient: 1 min, 1%B;5min,70%B;8min,70%B;9.5min,1%B;12min, 1% B. The flow rate was 0.7 ml/min. The mass spectrometer was operated in positive electrospray ionization (ESI) mode. The multiple reaction monitoring (MRM) parameters were set as follows: declustering potential set at 180 V for furmonertinib, 110 V for AST5902 (main active metabolite for furmonertinib), 80 V for osimertinib, 200 V for AZ5104 (main active metabolite for osimertinib) and 70 V for aumolertinib, collision energy set at 35 eV for furmonertinib, 34 eV for AST5902, 30 eV for osimertinib, 28 eV for AZ-5104 and 33 eV for aumolertinib, MRM transition set as m/z 569.5 → 411.2 for furmonertinib, m/z 555.5 → 498.5 for AST5902, m/z 500.8 → 455.3 for osimertinib, m/z 486.3 → 441.0 for AZ5104, m/z 526.5 → 481.3 for almonertinib (IS).

**Statistical analysis**

All preclinical data were presented as mean ± standard error of the mean (SEM). Statistical analyses were conducted using GraphPad Prism 9 software. For cell viability assays (n=6), each continuous variable was assessed for normal distribution using the Kolmogorov–Smirnov test. Group differences were evaluated with a two-tailed, unpaired t-test, assuming equal variances (F >0.05) and homogeneity of variance. For the pharmacokinetic profile (n=4), normality of continuous variables was assessed using the Shapiro–Wilk test, and group differences were analyzed with one-way ANOVA if F was greater than 0.05 and variance homogeneity was confirmed. Statistical significance was defined as ns, no significance, **p*<0.05, ***p*<0.01, ****p*<0.001.

**Supplementary Tables**

**Table S1 Summary of treatment-related adverse events (N=62)**

|  | **All grades(%)** | **Grade 3 or worse (%)** |
| --- | --- | --- |
| TRAEs | 49 (79) | 8 (12.9) |
| Rash | 14 (22.6) | 2 (3.2) |
| Diarrhea | 7 (11.3) | 1 (1.6) |
| Vomiting | 5 (8.1) | 3 (4.8) |
| Nausea | 3 (4.8) | 1 (1.6) |
| Hypertension | 3(4.8) | 1(1.6) |
| Fatigue | 4 (6.5) | 0 |
| Liver injury | 4 (6.5) | 0 |
| Proteinuria | 2 (3.2) | 0 |
| Oral ulcer | 2 (3.2) | 0 |
| Thrombocytopenia | 2 (3.2) | 0 |
| Blood bilirubin increased | 1 (1.6) | 0 |
| Leukopenia | 1 (1.6) | 0 |
| Gingivitis | 1 (1.6) | 0 |

Data are expressed as n (%). Abbreviations: TRAE, treatment-related adverse event.

**Supplementary Figures legends**

**Figure S1 Flowchart of the screening procedure.**

**Figure S2 Comparative pharmacokinetics of furmonertinib and Osimertinib.** (A) Plasma concentration-time profiles of furmonertinib, osimertinib, and their active metabolites. (B) Comparative plasma pharmacokinetic parameters of furmonertinib, osimertinib, and their active metabolites. (C) Brain concentration-time curves of furmonertinib, osimertinib, and their active metabolites. (D) Comparative brain pharmacokinetic parameters of furmonertinib, osimertinib, and their active metabolites. (E) Brain-to-plasma ratio at the time of peak drug concentration. All data were expressed as the mean ± SEM, ns, no significant, ** p<0.01 and *** p<0.001. N.D. not detected; N.A., not apply.

**Figure S3 Furmonertinib and its active metabolites showed no cross-resistance with Osimertinib.** (A) Establishment of an osimertinib-resistant EGFR-mutant non-small cell lung cancer cell line. (B) CCK-8 assay validated the growth inhibition of osimertinib on the sensitive PC9 cells and the resistant PC9/OR cells. (C-D) CCK-8 assay validated the growth inhibition by furmonertinib and its active metabolite AST5902 on the sensitive PC9 cells and the resistant PC9/OR cells. All of the data were expressed as the mean ± SEM, ns, no significant, * *p*<0.05, ** *p*<0.01, and *** *p*<0.001.
