# Supplementary figures and images for: Furmonertinib combined with bevacizumab in EGFR-TKI-resistant leptomeningeal metastasis: analysis of the CSF ctDNA molecular response and survival outcomes
Source: Br J Cancer. 2026 Apr 6;134(11):1614–23. doi: 10.1038/s41416-026-03407-z (PMC13184092; doi:10.1038/s41416-026-03407-z)

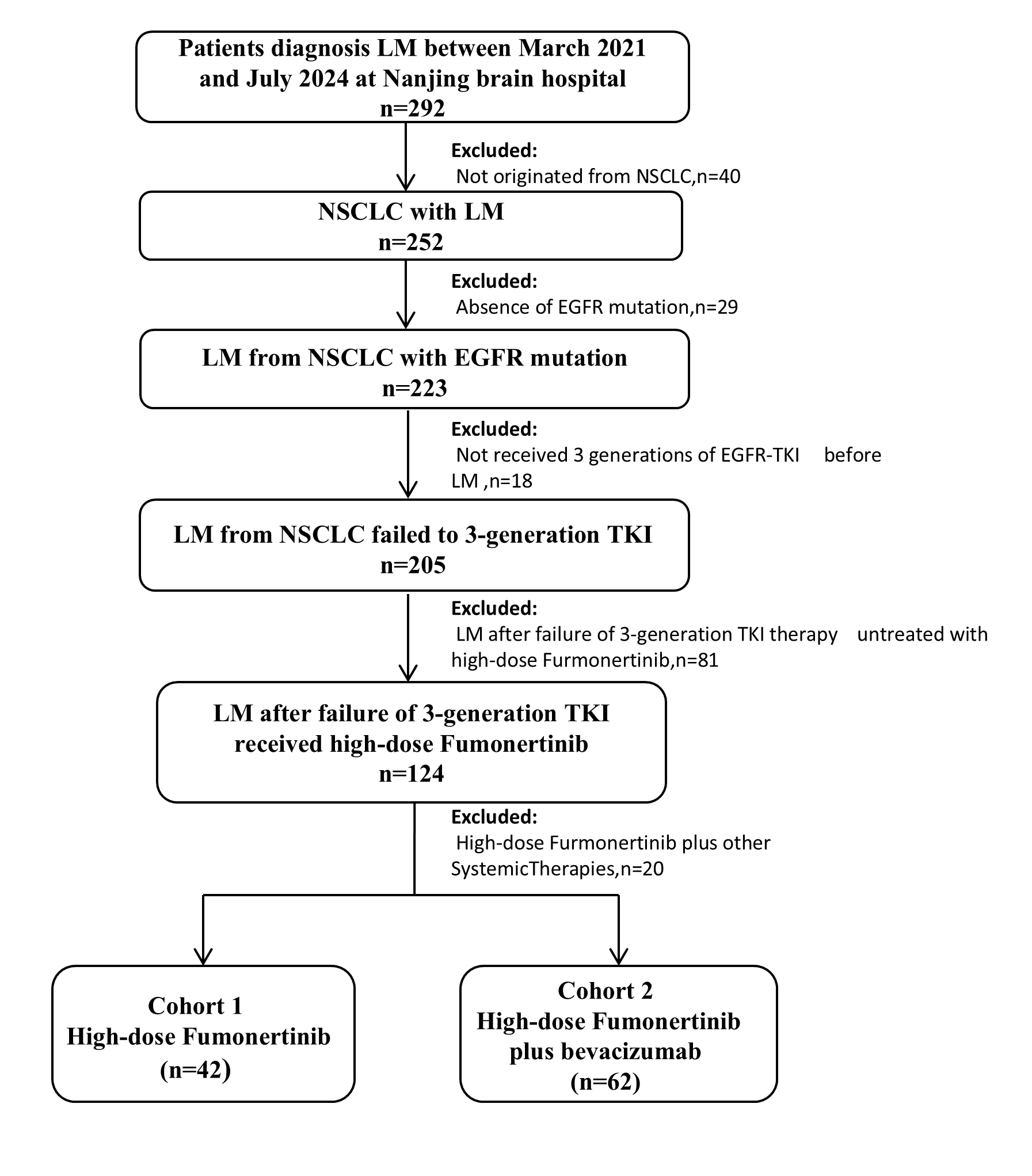

Supplement: Supplementary file 2 — Flowchart of the screening procedure. [file 41416_2026_3407_MOESM2_ESM.png]

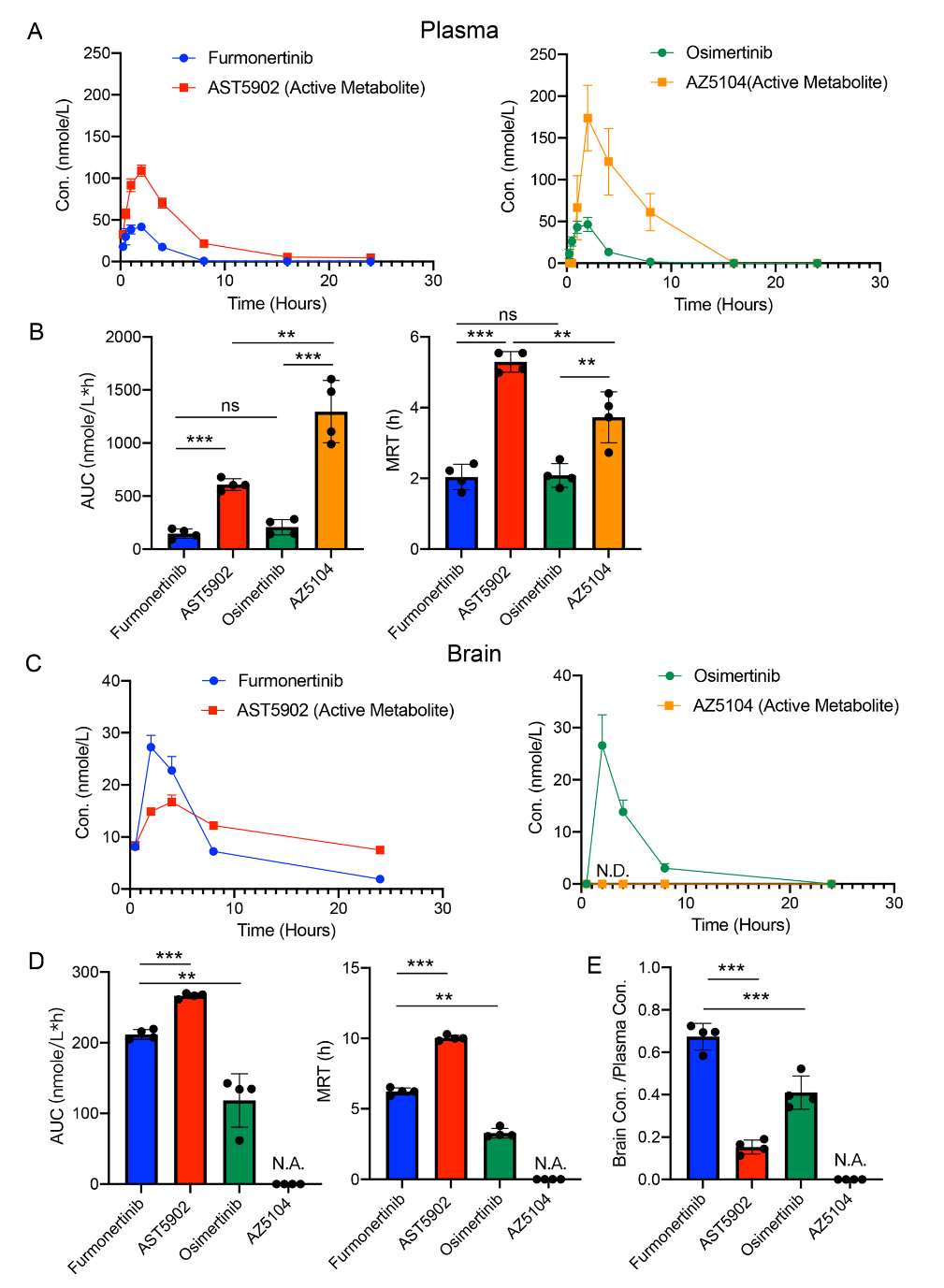

Supplement: Supplementary file 3 — Comparative pharmacokinetics of furmonertinib and Osimertinib. [file 41416_2026_3407_MOESM3_ESM.tif]

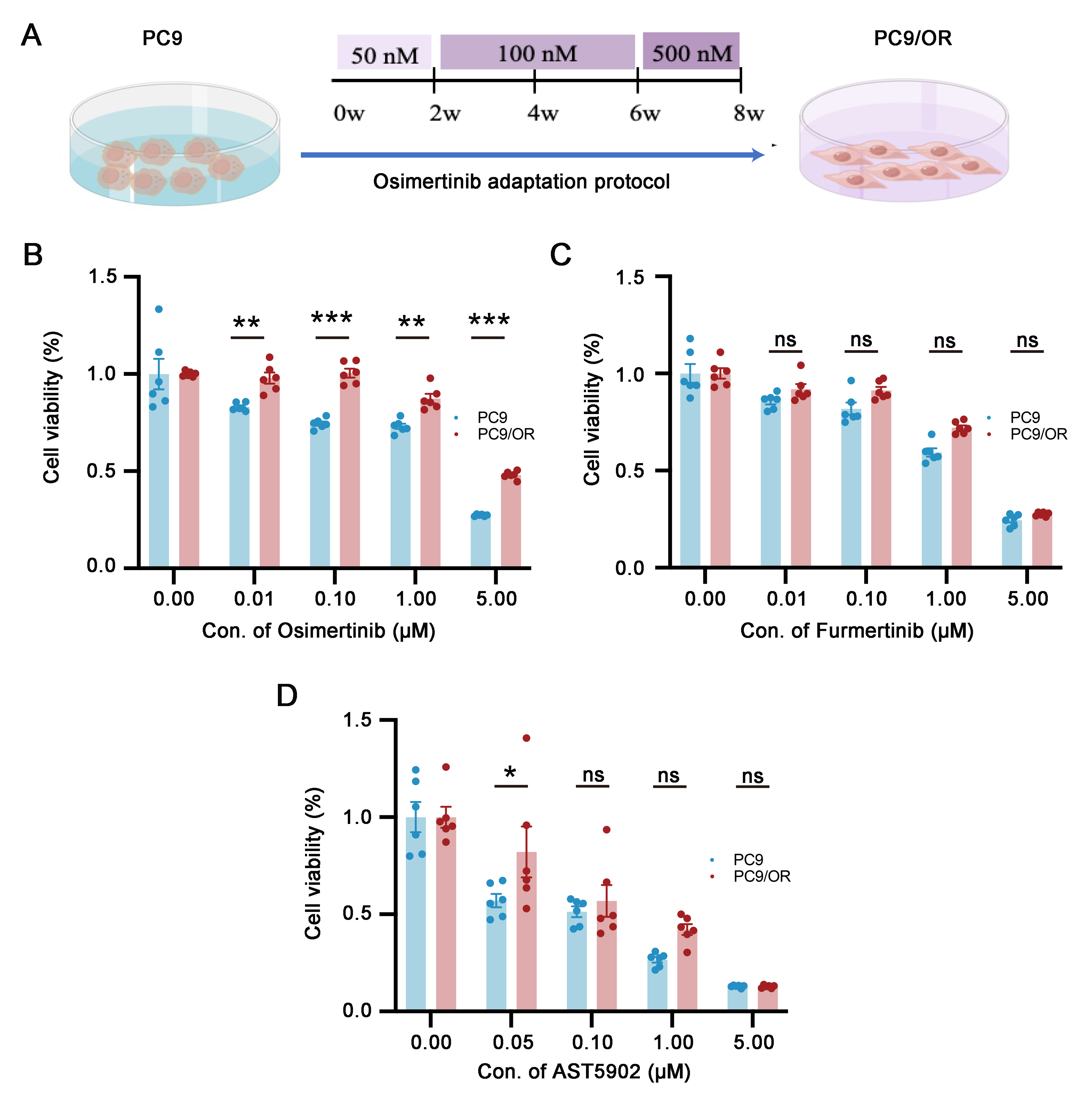

Supplement: Supplementary file 4 — Furmonertinib and its active metabolites showed no cross-resistance with Osimertinib. [file 41416_2026_3407_MOESM4_ESM.tif]
